# Prion protein polymorphisms associated with reduced CWD susceptibility limit peripheral PrP^CWD^ deposition in orally infected white-tailed deer

**DOI:** 10.1186/s12917-019-1794-z

**Published:** 2019-02-04

**Authors:** Alicia Otero, Camilo Duque Velásquez, Chad Johnson, Allen Herbst, Rosa Bolea, Juan José Badiola, Judd Aiken, Debbie McKenzie

**Affiliations:** 1 Centro de Encefalopatías y Enfermedades Transmisibles Emergentes, IA2, IIS, Universidad de Zaragoza, Zaragoza, Spain; 2 Department of Agricultural, Food and Nutritional Sciences, University of Alberta, Edmonton, Alberta Canada; 3 Department of Medicine, School of Medicine and Public Health, University of Wisconsin, Madison, USA; 4 Department of Biological Sciences, University of Alberta, Edmonton, Alberta Canada; 5 Centre for Prions and Protein Folding Diseases, Edmonton, Alberta Canada

**Keywords:** Prions, Prion diseases, Chronic wasting disease, CWD, PrP^CWD^, Peripheral tissues, Polymorphisms, Deer

## Abstract

**Background:**

Chronic wasting disease (CWD) is a prion disease affecting members of the *Cervidae* family. PrP^C^ primary structures play a key role in CWD susceptibility resulting in extended incubation periods and regulating the propagation of CWD strains. We analyzed the distribution of abnormal prion protein (PrP^CWD^) aggregates in brain and peripheral organs from orally inoculated white-tailed deer expressing four different *PRNP* genotypes: Q95G96/Q95G96 (wt/wt), S96/wt, H95/wt and H95/S96 to determine if there are substantial differences in the deposition pattern of PrP^CWD^ between different *PRNP* genotypes.

**Results:**

Although we detected differences in certain brain areas, globally, the different genotypes showed similar PrP^CWD^ deposition patterns in the brain. However, we found that clinically affected deer expressing H95 PrP^C^, despite having the longest survival periods, presented less PrP^CWD^ immunoreactivity in particular peripheral organs. In addition, no PrP^CWD^ was detected in skeletal muscle of any of the deer.

**Conclusions:**

Our data suggest that expression of H95-PrP^C^ limits peripheral accumulation of PrP^CWD^ as detected by immunohistochemistry. Conversely, infected S96/wt and wt/wt deer presented with similar PrP^CWD^ peripheral distribution at terminal stage of disease, suggesting that the S96-PrP^C^ allele, although delaying CWD progression, does not completely limit the peripheral accumulation of the infectious agent.

## Background

Chronic wasting disease (CWD) is a transmissible spongiform encephalopathy (TSE) of cervids, the only known TSE found in both farmed and free-ranging animals [1, 2]. Like other TSEs, CWD is a fatal neurodegenerative disease caused by the conversion of the host-encoded cellular prion protein (PrP^C^) to a misfolded isoform (PrP^CWD^) through templated or seeded polymerization mechanisms [3–5]. CWD affects cervid populations in North America, South Korea and Northern Europe [6]. Horizontal transmission by direct animal interactions and via persistence of infectivity in the environment hinders control and eradication of these diseases [7–10].

It has been widely documented that certain polymorphisms of the prion protein gene (*PRNP*), encoding PrP^C^, play a key role in the susceptibility to prion diseases [11–14]. A close relationship between *PRNP* variability and CWD infection status in wild cervids has also been described [15–17]. The prevalence of CWD is lower in white-tailed deer expressing at least one copy of the H95 or S96 polymorphisms suggesting reduced susceptibility to infection [17]. The direct effect of these polymorphisms on disease progression was evaluated through experimental oral infection studies where CWD source, dose and route of infection were controlled. This experimental infection demonstrated that H95 and S96 polymorphisms impact CWD progression since deer homozygous for the wild-type (wt) alleles (Q95/G96) presented shorter incubation periods and a more rapid clinical disease phase than deer expressing at least one copy of H95 or S96 alleles [18]. In addition to greatly increasing the survival period of deer orally challenged with wt-CWD prions [18], the H95 allele modulated the emergence of a novel prion strain H95^+^, which possesses singular biochemical and biological properties [19, 20].

Together with the infecting strain, the *PRNP* genotype is a major factor influencing the neuropathological phenotype [21–24]. Although less studied, variability at *PRNP* may also affect the pathways of neuroinvasion and the involvement of other tissues [25, 26]. In sheep scrapie, the expression of arginine at position 171 has profound repercussions on PrP^Sc^ replication and distribution [13, 27–29]. R171 heterozygous sheep show lower accumulation of PrP^Sc^ in the lymphoreticular system (LRS) and other tissues as compared to Q171 homozygous sheep [25, 30]. Thus, PrP^C^ polymorphisms might also have an effect on tissue-specific PrP^CWD^ accumulation. In CWD, it has been observed that PrP^CWD^ deposition in the brain and other organs progress at a slower rate in deer expressing polymorphisms associated with a lower frequency of CWD natural cases [17, 26, 31]. However, observations are often made in free-ranging, naturally infected animals, which limit the conclusions that can be obtained about the potential effect of the genotype on PrP^CWD^ deposition, due to the variability in the infecting strains, routes of exposure and incubation periods.

Using immunohistochemistry (IHC), we evaluated PrP^CWD^ deposition in orally inoculated white-tailed deer expressing different *PRNP* genotypes: wt/wt, S96/wt, H95/wt and H95/S96 [18] including a thorough characterization of PrP^CWD^ distribution in the nervous system, lymph system and peripheral organs. We observed that deer expressing H95 PrP^C^ accumulated less PrP^CWD^ in peripheral organs at terminal stage of the disease.

## Results

### PrP^CWD^ deposition in lymphoid tissues and nervous system

PrP^CWD^ deposition was detected by immunohistochemistry in lymphoid tissues and the brain from all clinically affected deer regardless of *PRNP* genotype. PrP^CWD^ deposits appeared as bright-red granular material in Peyer’s patches, tonsils, spleen and lymph nodes from CWD-challenged deer. In general, PrP^CWD^ immunolabeling was more intense in the lymph nodes of the head and visceral lymph nodes, whereas lymph nodes of the limbs (prescapular, axillary, prefemoral, popliteal and inguinal) showed a lower number of positive follicles and milder immunostaining in all deer. Consistently with these observations, one S96/wt animal (D8) showed no PrP^CWD^ deposition in axillary, prescapular, prefemoral and inguinal lymph nodes. Lymphoid follicles of third eyelid and rectal mucosa were strongly PrP^CWD^ positive when the histological sample contained follicles that allowed immunohistochemical analysis (Table 1).

**Table 1:** Distribution of PrP^CWD^ deposits in lymphoid tissues of clinically affected and non-inoculated white-tailed deer

											Non-inoculated	
Genotype	wt/wt					S96/wt			H95/wt	H95/S96	wt/wt	
	D1	D2	D3	D4^a^	D5	D6	D7	D8	D9	D10	D11	D12
Retropharyngeal LN	++	+++	+++	/	++	++	++	+++	++	+++	–	–
Submandibular LN	+++	++	++	/	+++	++	++	++	/	/	–	–
Axillary LN	++	++	+	/	+++	++	++	–	++	+	–	–
Prescapular LN	+	+	+	/	++	+	–	–	+	++	–	–
Prefemoral LN	++	+	++	/	+	+	+	–	+	++	–	–
Popliteal LN	+	+	++	/	+++	+	+	+	+	+	–	–
Inguinal LN	+	+	++	/	+++	+	+	–	/	++	–	–
Tracheobronchial LN	++	+	+++	/	+++	/	/	/	/	/	–	–
Ileocecal LN	/	++	++	/	+++	++	+++	++	++	+++	–	–
Hepatic LN	/	+++	/	/	/	++	++	+++	+++	+++	/	/
Pancreatic LN	+++	/	/	/	/	++	+++	/	/	/	–	/
Adrenal LN	+++	++	/	/	–	++	+++	++	/	++	/	–
Spleen	+	+	+	+	+	–	+	+	+	+	–	–
3rd eyelid	+	+	++	/	-^b^	- ^b^	- ^b^	+	/	+	–	–
Tonsil	+++	+++	+++	+++	+++	+++	+++	+++	+++	+++	–	–
Peyer’s Patches	+++	+++	+++	+++	+++	+++	+++	/	+++	+++	–	–
Rectal lymphoid follicles	+++	+++	+++	+++	++	+++	+	/	/	+++	–	–

PrP^CWD^ deposition was detected by IHC

^a^No lymph nodes were collected from D4 ^b^No lymphoid follicles were present in the histological sample. / Sample not available for evaluation

**Fig. 1:**
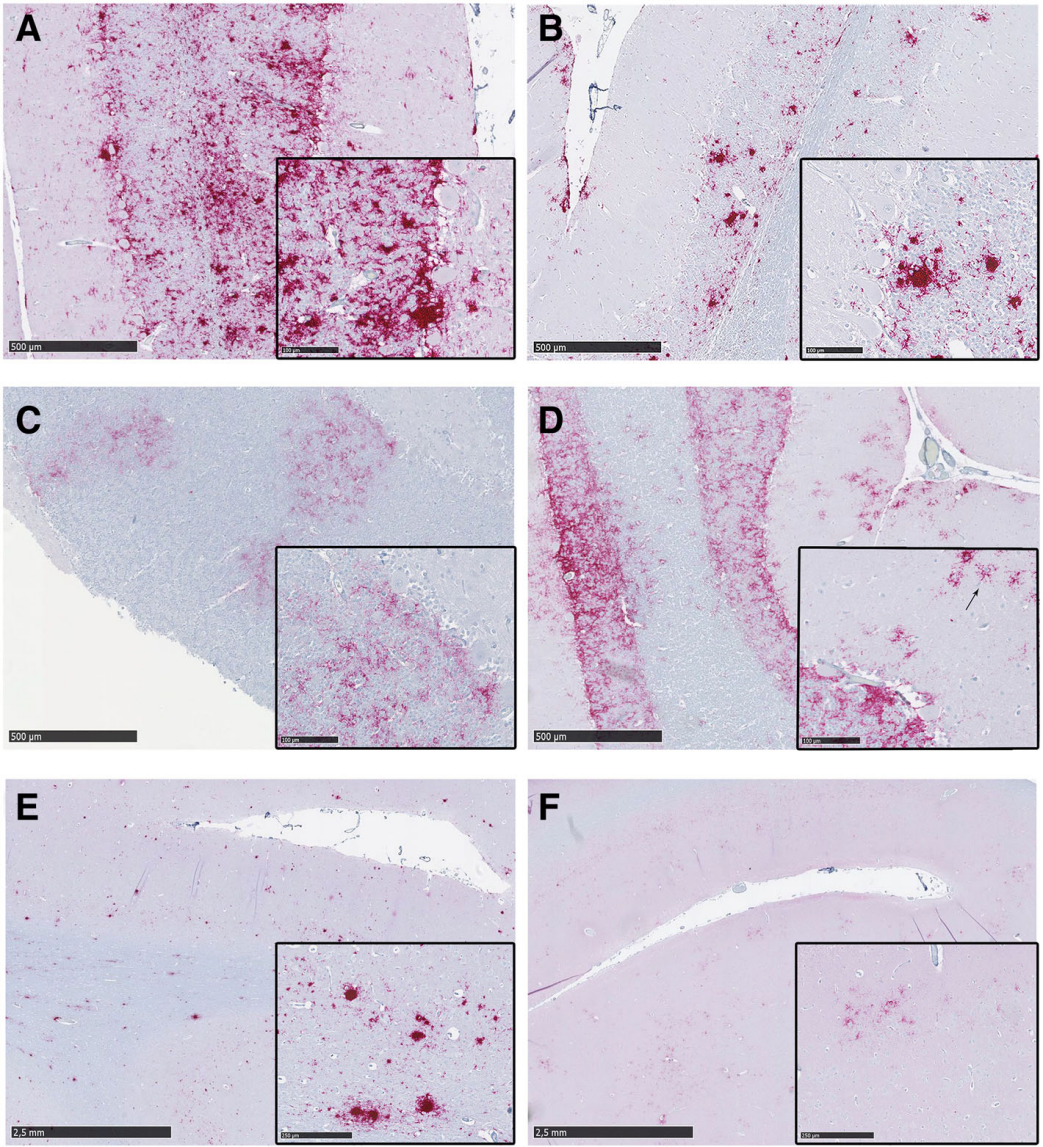
PrP^CWD^ deposition pattern in the cerebellum (**a** to **d**) and the frontal cortex (**e**, **f**) of clinically affected deer. Inserts contain magnified images of the corresponding sample to show the morphology and limits of the PrP^CWD^ aggregates. **a** Cerebellum of a wt/wt deer showing abundant coalescing PrP^CWD^ deposits and plaques in the granular and Purkinje cell layer. **b** S96/wt deer showing evident milder deposition. PrP^CWD^ plaques are observed only in the granular layer. **c** Cerebellum from the H95/wt deer showing a patch-shaped distribution of coarse granular and fine punctate PrP^CWD^ aggregates through the granular layer. **d** Cerebellum from the H95/S96 deer, which presented coarse granular aggregates homogeneously distributed through the granular layer and stellate aggregates in the molecular layer (arrow). **e** Frontal cortex from a wt/wt deer showing abundant plaques in grey and white matter. **f** Frontal cortex from the H95/S96 deer showing mild deposition of fine punctate cell-associate aggregates

Brain samples presented intense PrP^CWD^ immunolabeling in all deer genotypes. The PrP^CWD^ profile was characterized by plaques and coarse granular and coalescing extracellular deposits mainly located around neurons, glial cells, vacuoles and along myelinated axons of the white matter (Fig. 1). Although less frequent, intraneuronal PrP^CWD^ deposition was also observed, especially in the dorsal motor nucleus of the vagus nerve, the hypoglossal nucleus, the spinal trigeminal nucleus and the inferior olivary nucleus of the obex of all clinical deer.

Surprisingly, each *PRNP* genotype presented distinguishable PrP^CWD^ pathological phenotypes in the cerebellum. Wt/wt deer showed severe PrP^CWD^ immunostaining in granular layer with coarse granular and large plaques invading the Purkinje cell layer and extending to the molecular layer (Fig. 1a). PrP^CWD^ plaques were also present in the cerebellum of all S96/wt clinically affected deer. However, for animals of this genotype, the presence of plaques was restricted to granular layer and white matter, whereas the Purkinje cell and the molecular layer showed milder granular and diffuse PrP^CWD^ deposits compared to wt/wt deer (Fig. 1b). Conversely, the cerebellar pathological phenotype of the H95/wt deer was characterized by discontinuous and diffuse PrP^CWD^ labeling in the granular layer, showing predominantly fine punctate and coarse small granular deposits (Fig. 1c), although a few plaque-like deposits were also observed. Finally, the cerebellum of the H95/S96 deer showed fine punctate and coarse granular PrP^CWD^ deposits homogeneously distributed through the granular layer. The cerebellar molecular layer of this deer presented a more intense immunolabeling than deer of other genotypes, showing conspicuous stellate PrP^CWD^ aggregates (Fig. 1d).

In the frontal cortex, the H95/S96 deer PrP^CWD^ morphological profile differed from that observed in the other deer genotypes. All wt/wt, S96/wt and the H95/wt deer presented abundant coalescing deposits and large PrP^CWD^ plaques in both grey and white matter (Fig. 1e). H95/S96 deer, however, had milder staining in the frontal cortex, presenting coarse and diffuse granular, cell-associated aggregates mostly confined to grey matter (Fig. 1f), whereas white matter deposits were sparse, and predominantly of the perivascular type.

**Fig. 2:**
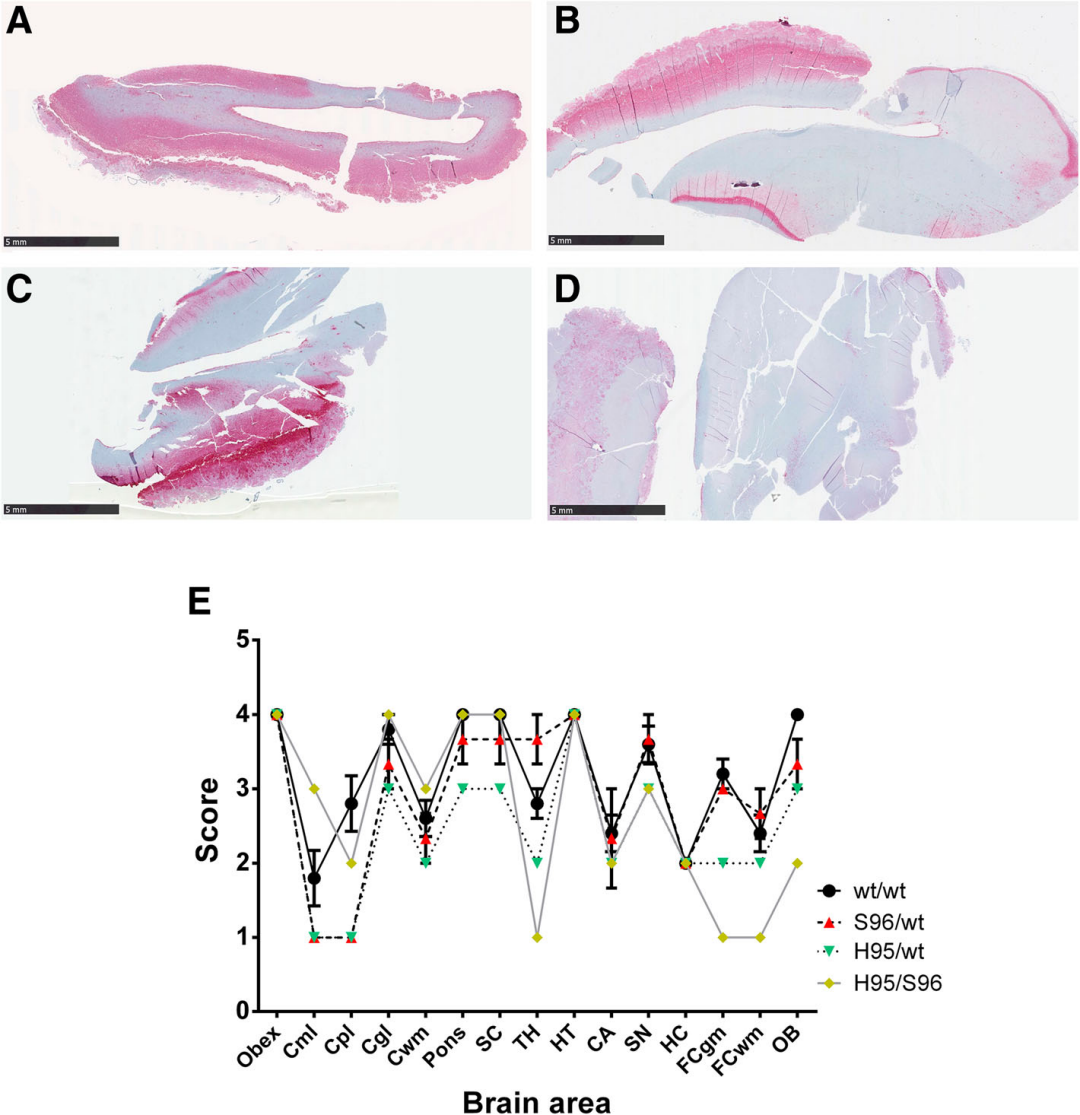
PrP^CWD^ deposition in the brain of CWD infected deer. **a** Representative olfactory bulb sample from a wt/wt deer showing abundant PrP^CWD^ immunolabeling diffusely distributed in the brain area. **b** Olfactory bulb sample from a S96/wt and the (**c**) H95/wt deer showing similar PrP^CWD^ immunolabeling, more restricted to the grey matter. **d** Olfactory bulb sample from the H95/S96 deer showing mild PrP^CWD^ immunolabeling. **e** PrP^CWD^ deposition profile of the experimentally infected deer of different *PRNP* genotypes. Evaluated brain areas are: Obex; Cml, cerebellar molecular layer; Cpl, cerebellar Purkinje cell layer; Cgl, cerebellar granular layer; Cwm, cerebellar white matter; Pons; SC, superior colliculus; TH, thalamus; HT, hypothalamus; CA, caudate nucleus; SN, septal nucleus; HC, hippocampus; FCgm, frontal cortex grey matter; FCwm, frontal cortex white matter; OB, Olfactory bulb

PrP^CWD^ neuroanatomical distribution was similar for all deer irrespective of their *PRNP* genotypes. Intense staining was observed in obex, superior colliculus, hypothalamus, septal nucleus of the basal ganglia and cerebellar granular layer in all deer (Fig. 2e). Some differences however, were observed between these deer. Compared to deer of other genotypes, H95/S96 deer showed reduced PrP^CWD^ immunoreactivity in thalamus, frontal cortex and olfactory bulb (Fig. 2). The milder immunolabeling observed in H95/S96 deer may explain the different PrP^CWD^ morphological features found in frontal cortex (Fig. 1f).

Optic nerves presented PrP^CWD^-specific staining in all clinically affected deer. In the rest of the peripheral nerves, PrP deposition was irregular. The vagus nerve was the most consistent, with chromagen granules observed in 5 of the 6 evaluated samples. Only a few aggregates were detected in 2 of the 8 sciatic nerves evaluated and the brachial plexus was negative for PrP^CWD^ accumulation in all deer (Table 2).

**Table 2:** Distribution of PrP^CWD^ deposits in peripheral nerves, glands and organs of clinically affected and non-inoculated white-tailed deer

											Non-inoculated	
											wt/wt	
Genotype	wt/wt					S96/wt			H95/wt	H95/S96		
	D1	D2	D3	D4	D5	D6	D7	D8	D9	D10	D11	D12
Optic nerve	++	+	++	+	+	+	/	+	+	++	/	–
Vagus nerve	++	+	+	/	/	+	–	+	–	+	–	–
Brachial plexus	/	–	–	/	/	–	–	/	/	–	–	–
Sciatic nerve	+	+	–	/	–	–	/	–	–	–	–	–
Pituitary gland	/	++	+++	/	/	+++	+++	+++	/	+++	–	–
Islets of Langerhans	++	++	++	++	++	++	+	+	–	–	/	–
Adrenal Gl medulla	+++	+++	+++	+++	++	+++	+++	+++	+	++	/	–
Ileoc. Valve villi/crypts	++	+	+	+	++	++	++	/	–	–	–	–
Kidney	++^a^	+	+	+	+	/	+	+	–	–	–	–
Parotid salivary gland	+	+	–	/	+	+	–	+	–	/	/	–
Submandibular sal. Gl	/	+	–	/	+	+	+	+	–	–	/	–
Sublingual sal. Gl	/	–	–	/	/	–	–	–	–	–	/	–
Retina	+++	+++	+++	+	+++	+++	+	+++	++	++	/	–
Skeletal muscle	–	–	–	–	–	–	–	–	–	–	–	–
Heart	/	/	/	/	/	++	/	/	–	–	/	/
Lung	+ ^a^	–	–	–	–	–	–	–	–	–	–	–
Liver	–	–	–	–	–	–	–	–	–	–	–	–

PrP^CWD^ deposition was detected by IHC

^a^Deer showing inflammatory kidney disease and lung interstitial inflammation / Sample not available for evaluation

### PrP^CWD^ deposition in endocrine tissues

**Fig. 3:**
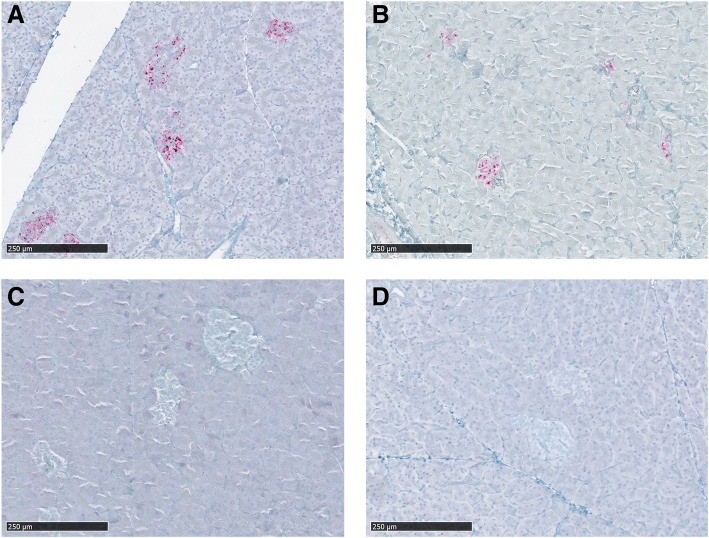
Immunohistochemical detection of PrP^CWD^ in the pancreas of CWD affected white-tailed deer. **a** Pancreas from a wt/wt deer and a (**b**) S96/wt deer showing PrP^CWD^ deposition in the islets of Langerhans. **c** Pancreas from the H95/wt and the (**d**) H95/S96 deer, which did not present any positive immunolabeling

Pituitary gland was collected from six clinically affected deer, two wt/wt, three S96/wt and the H95/S96 deer. This gland presented abundant PrP^CWD^ deposition in all deer, especially affecting *pars nervosa* and *pars intermedia*, although PrP^CWD^ staining was also present in *pars distalis*. In *pars distalis*, PrP^CWD^ staining was mild and scattered, and found in the connective tissue framework. In addition, PrP^CWD^ immunolabeling was observed in the pancreata of clinically affected wt/wt and S96/wt deer, restricted in the islets of Langerhans (Table 2 and Fig. 3a, b) which are scattered clusters of endocrine cells. In these animals, positive islets of Langerhans were abundant and often adjacent, as previously described for CWD [32]. Interestingly, no PrP^CWD^ deposits were observed in pancreatic tissues of either the H95/wt or H95/S96 deer (Table 1 and Fig. 3c, d).

Adrenal glands were also positive by IHC in all clinically affected genotypes. Although the most abundant immunolabeling was detected in the adrenal medulla, which presented a pattern of immunoreactivity similar to that previously described in other prion diseases [33], positive immunolabeling was also detected in the adrenal cortex. In adrenal cortex, granular and scant immunopositive deposits were found in the innermost zone (*zona reticularis*). The presence of immunopositive material in the adrenal cortex was more abundant in the group of wt/wt deer.

### PrP^CWD^ deposition in muscle

**Fig. 4:**
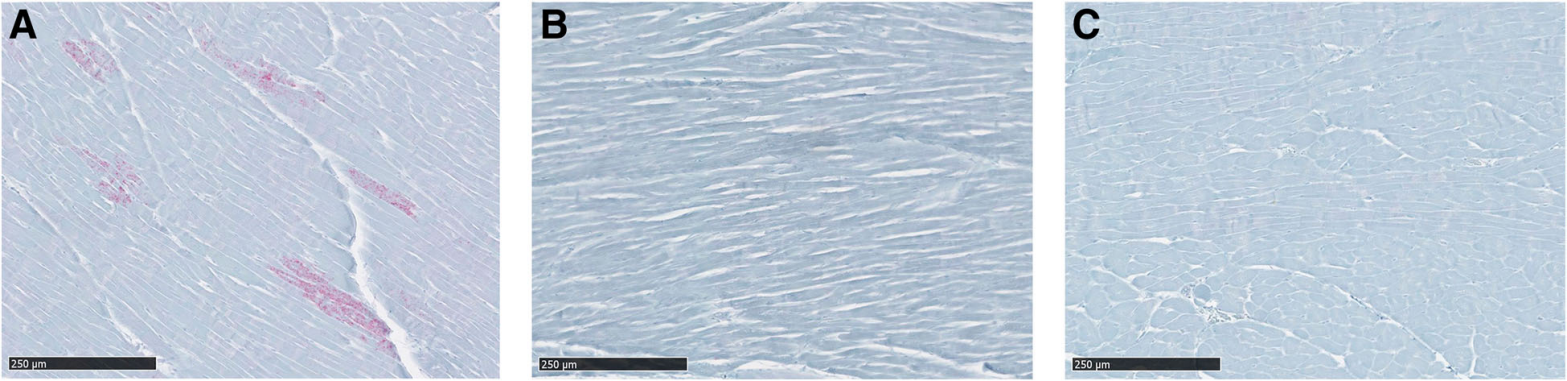
Immunohistochemical detection of PrP^CWD^ in the heart of CWD affected white-tailed deer. **a** Heart from a S96/wt deer showing positive immunolabeling in separated groups of cardiac myocytes. **b** Heart from the H95/wt and the (**c**) H95/S96 deer in which no positive immunolabeling was detected

Skeletal muscle samples were collected from tongue as well as forelimb and hindlimb muscles of the CWD challenged deer. No PrP^CWD^ immunolabeling was detected within skeletal muscle tissues. PrP^CWD^ deposits were neither observed in muscle-associated nerve fascicles, structures that have been previously reported to show PrP^CWD^ accumulation in CWD infected white-tailed deer [34]. Immunoreactivity was also not detected in neuromuscular spindles; structures in which prion deposition has previously been reported in sheep scrapie [33, 35]. Heart samples collected from a S96/wt deer (D6) showed immunolabeling. In this S96/wt deer, we observed scattered PrP^CWD^ aggregates affecting separated groups of cardiac myocytes (Fig. 4a). In addition, PrP^CWD^ immunolabeling was more visible in longitudinal cross-sections of the cardiac muscle, an accumulation pattern similar to that previously described in the heart of white-tailed deer infected with CWD [36]. Surprisingly, all cardiac muscle samples from H95/wt and H95/S96 deer lacked detectable PrP^CWD^ immunoreactivity (Fig. 4b, c).

### PrP^CWD^ deposition in intestinal tract

**Fig. 5:**
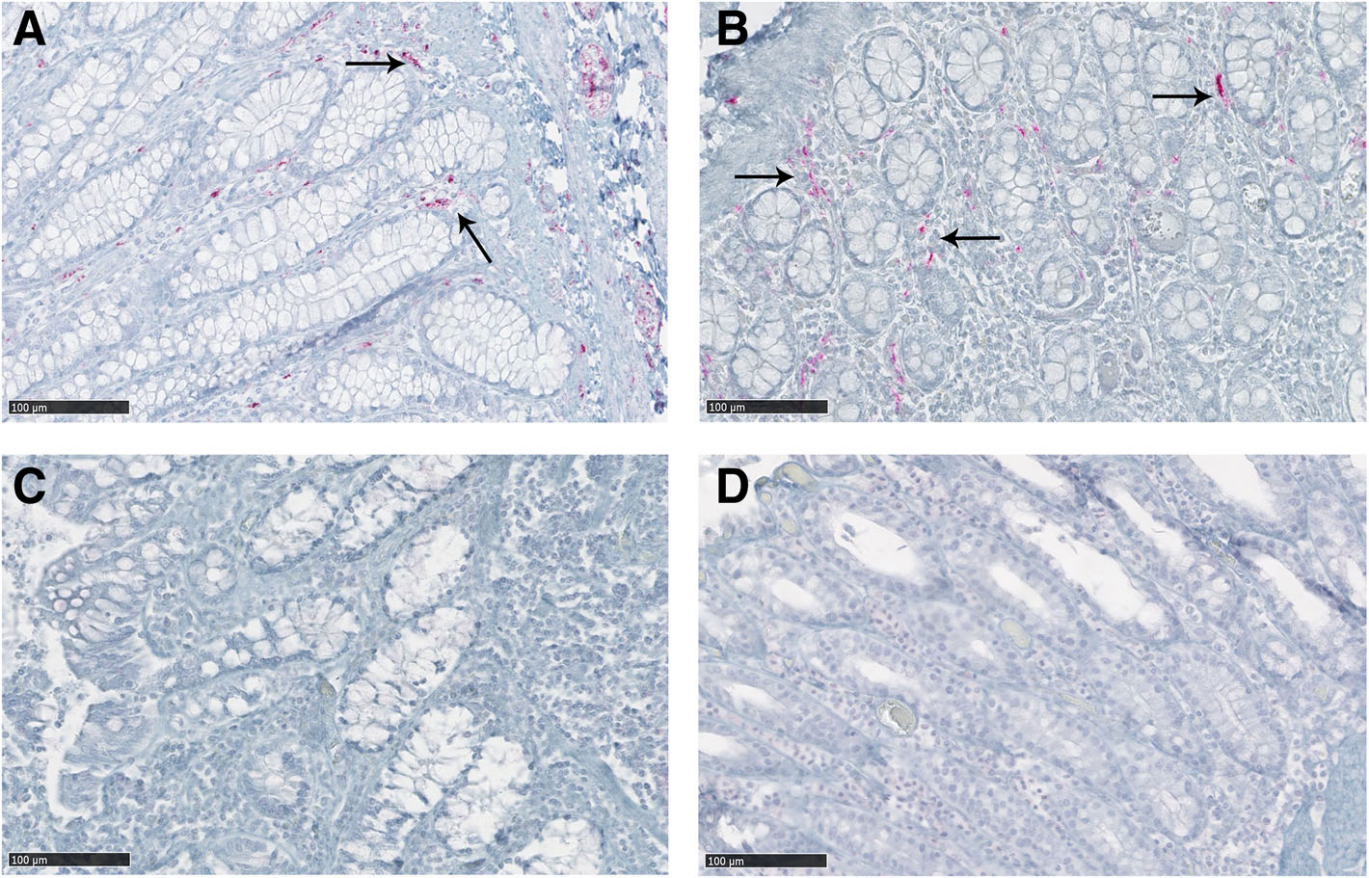
PrP^CWD^ deposition in the crypts of the ileocecal junction of CWD affected white-tailed deer. **a** Ileocecal junction mucosa from a wt/wt deer and a (**b**) S96/wt deer showing PrP^CWD^ immunolabeling dispersed along the lamina propria between intestinal crypts (arrows). This PrP^CWD^ immunolabeling pattern was not observed in the ileocecal junction of the (**c**) H95/wt nor the (**d**) H95/S96 deer

Gut-associated lymphoid and nervous tissues accumulated high levels of PrP^CWD^ in all clinically positive CWD-infected deer. IHC positive material was more abundant in Peyer’s patches and nerve fibers and ganglia of the enteric nervous system (ENS) in all intestinal segments evaluated. However, differences in the distribution of PrP^CWD^ in the intestinal tract were evident between genotypes. The H95/wt and H95/S96 deer presented reduced accumulation of PrP^CWD^ in the villi and crypts of the intestinal mucosa compared to wt/wt and S96/wt deer. Differences were most noticeable at the ileocecal junction, with all wt/wt and S96/wt deer showing strong PrP^CWD^ deposition dispersed along the lamina propria between villi and crypts, while H95/wt and H95/S96 deer showed no immunolabeling (Fig. 5).

### PrP^CWD^ deposition in kidney

**Fig. 6:**
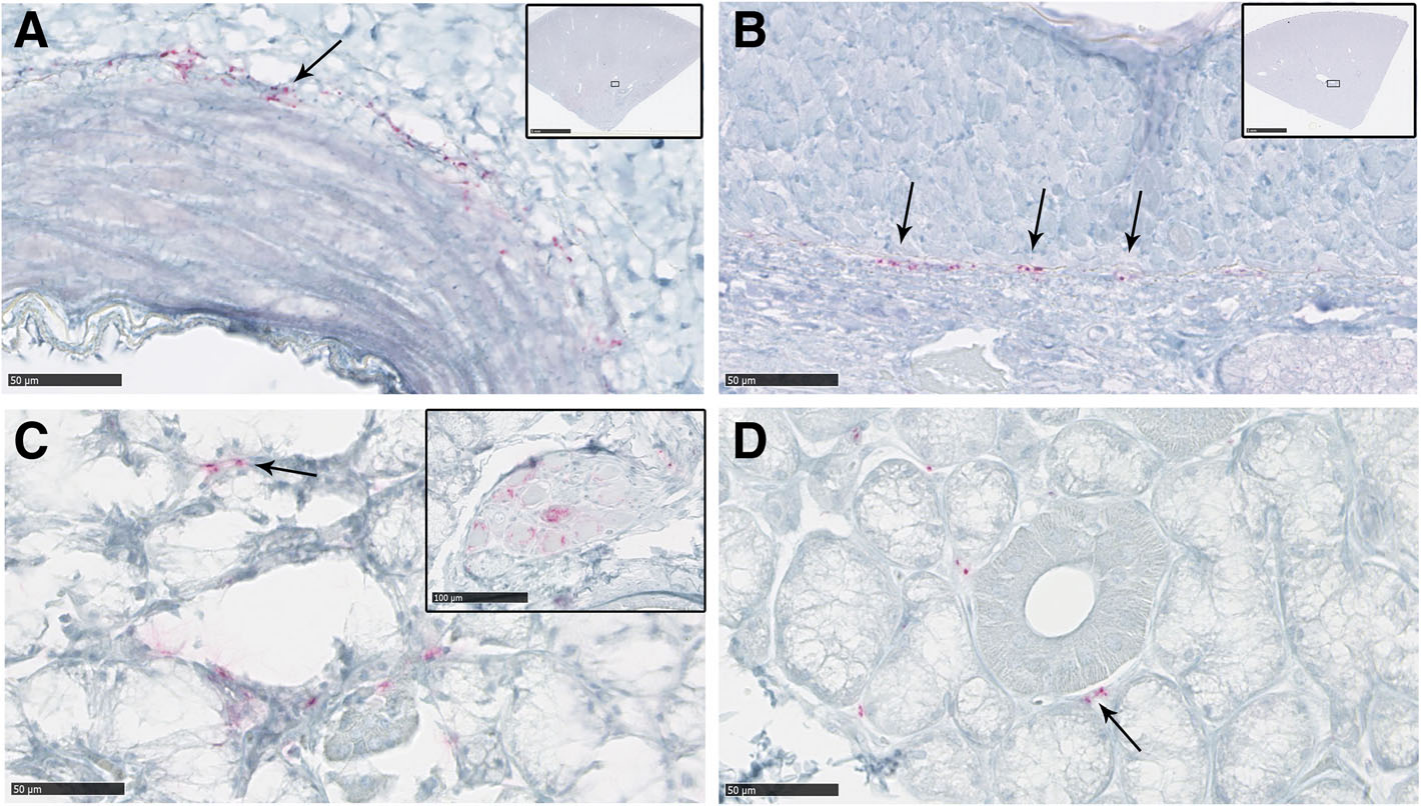
PrP^CWD^ deposition in the kidney and salivary glands of clinically-affected white-tailed deer. **a** Kidney from a wt/wt deer and a (**b**) S96/wt deer showing periarterial PrP^CWD^ deposition in arcuate arteries (arrows). Inserts show the specific location of these arteries in the histopathological sample. **c** Salivary gland from a wt/wt deer showing positive PrP^CWD^ immunolabeling in the interstitial tissue between acini (arrows). PrP^CWD^ immunolabeling was also detected in the ganglion neurons immersed in the salivary gland sample (insert picture). **d** Salivary gland from a S96/wt deer. Positive immunolabeling was detected in the same location as for wt/wt deer (arrow). No PrP^CWD^ deposition was observed in the kidneys or salivary glands of deer expressing the H95-PrP^C^

**Fig. 7:**
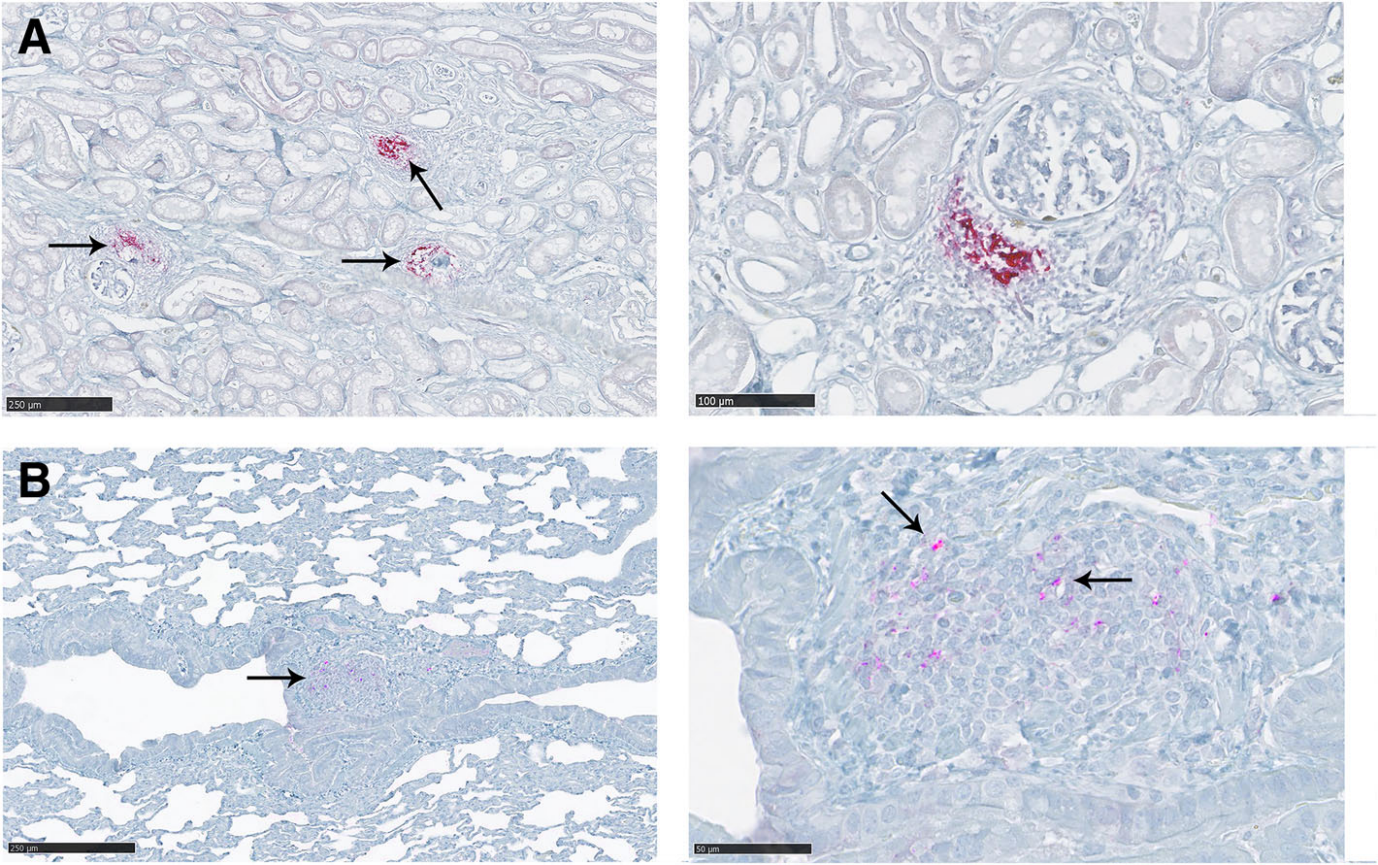
PrP^CWD^ deposition in the kidney (**a**) and the lung (**b**) of a deer presenting signs of inflammation (D1). **a** A strong PrP^CWD^ deposition associated with foci of inflammatory cells was observed in this deer. Those foci of inflammation were generally found in the proximity of the glomeruli. **b** A moderate interstitial inflammation was also detected in this animal. PrP^CWD^ accumulation was observed associated with inflammatory cells (arrows)

Kidney samples were evaluated from 9 of the 10 CWD clinical deer. Positive immunolabeling was found in all evaluated kidney samples from wt/wt and S96/wt deer. PrP^CWD^ staining was consistently associated with arterial vessels, showing a periarterial and periarteriolar deposition. The most abundant immunolabeling was detected in the wall of the main renal artery and arcuate arteries, which locate at the junction of the renal cortex and the renal medulla and arise from interlobar arteries. Interestingly, no PrP^CWD^ deposits were found in any of the evaluated kidney samples from the H95/wt and the H95/S96 deer (Table 2, Fig. 6). In addition to the PrP^CWD^ aggregates observed in arterial vessels, one wt/wt deer had strong PrP^CWD^ deposition associated with foci of inflammatory cells (D1) affecting renal glomeruli, which was compatible with a moderate interstitial glomerulonephritis (Fig. 7a).

### PrP^CWD^ deposition in salivary glands

PrP^CWD^ deposits found in salivary glands were mild and scattered. Positive immunolabeling was found in the interstitial tissue between acini, whereas no intracellular deposits were detected in acinar cells or within salivary ducts in any of the samples evaluated. Parasympathetic ganglia neurons innervating the salivary gland tissue from a wt/wt deer (D4), presented strong intraneuronal immunolabeling (Fig. 6c). IHC deposits were observed in parotid and submandibular salivary glands, whereas all the evaluated sublingual glands samples were negative for PrP^CWD^ immunostaining. Deposition in the interstitial tissue was especially evident in the submandibular salivary gland of D7 (Fig. 6d). Positive immunolabeling was detected in at least one salivary gland sample from all clinically affected deer with the exception of H95/wt and H95/S96 deer, which showed no PrP^CWD^ deposits in any of the salivary glands evaluated.

### PrP^CWD^ deposition in other organs

PrP^CWD^ immunolabeling was detected in the lungs of D1. This deer showed mild lung interstitial inflammation and edema. PrP^CWD^ aggregates were observed to be associated with inflammatory cell foci and in the cells of the bronchiolar epithelium (Fig. 7b). This deer also showed, as mentioned previously, positive immunolabeling related to the accumulation of inflammatory cells in the kidney (Fig. 7a). Eye samples were collected from all deer included in this study. In addition to the positive immunolabeling of the optic nerve, described above, all deer were also positive for PrP^CWD^ IHC deposition in the retina. All collected liver samples from the clinically affected deer were IHC negative (Table 2).

Corresponding negative tissue controls from uninfected deer were included when available. These tissues did not present any immunolabeling, confirming the specificity of the immunohistochemical detection (Tables 1 and 2).

## Discussion

We found that the intensity and distribution of PrP^CWD^ deposits in brain and peripheral tissues of *PRNP* polymorphic (i.e. different PrP^C^ primary structures) white-tailed deer was distinct from Q95G95 (wt) homozygous deer exposed to the same prion strain (i.e. Wisc-1). We have previously shown that H95 and S96 *PRNP* polymorphisms play a key role in CWD susceptibility, increasing survival periods and having dramatic effects on the propagation of CWD strains [16–20, 37].

Our results show that deer expressing the H95-PrP^C^ presented a more limited peripheral distribution of PrP^CWD^ compared to wt/wt and S96/wt deer. Under identical experimental conditions and disease stage, the number of organs with positive immunolabeling was reduced in deer with H95-PrP^C^ allelotypes [18]. The most significant differences in PrP^CWD^ deposition between deer with different *PRNP* genotypes were found in pancreas, heart, kidney and intestine samples. Both deer expressing the H95-PrP^C^ showed no immunolabeling or reduced accumulation of PrP^CWD^ aggregates in these tissues.

The presence of PrP^CWD^ in endocrine tissues has been previously described in the adrenal medulla, the pituitary gland and islets of Langerhans in the pancreas of CWD-affected cervids [31, 32], results that agree with our observations in CWD-infected wt/wt and S96/wt deer. Only deer of these genotypes (Fig. 3a, b) showed moderate to strong chromagen deposition in islets of Langerhans, which are innervated by the vagus nerve [32]. Differences in adrenal glands immunolabeling were minor between deer expressing different PrP^C^ polymorphisms. Consistent with wt-PrP^C^ being the cognate substrate for Wisc-1 homologous prion conversion, wt/wt deer presented higher and widespread PrP^CWD^ deposition compared to deer of other genotypes.

The distribution of PrP^CWD^ aggregates was also limited in hearts of animals expressing the H95-PrP^C^ allelotype. Cardiac tissues were collected from D6 (S96/wt), D9 (H95/wt) and D10 (H95/S96). PrP^CWD^ accumulation was detected in multiple heart samples from D6, affecting separated groups of cardiac myocytes (Fig. 4a). Conversely, no immunolabeling was observed in any heart sample from deer expressing the H95-PrP^C^ (Fig. 4b, c).

A distinct pattern of distribution of PrP^CWD^ was also observed in intestinal tissues for H95 carriers. It is not surprising that strong immunolabeling was observed in gut-associated lymphoid and nervous tissues as these are known to be the first sites of PrP^d^ accumulation following oral infection [26, 30, 31, 38]. Nevertheless, H95/wt and H95/S96 deer, which had the longest incubation periods [18], showed more restricted or localized PrP^CWD^ accumulation (Fig. 5). These findings however, do not necessarily indicate that these animals excrete a lower amount of prions or present a lack of infectivity in intestinal tissues.

PrP^CWD^ deposits were observed in renal tissues of all wt/wt and S96/wt deer evaluated in the present study, whereas no immunopositive material was found in any of the kidney samples collected from deer expressing the H95 allele (Fig. 6). Immunohistochemical detection of PrP^CWD^ in kidneys of CWD-infected white-tailed deer has previously only been reported in ectopic lymphoid follicles [31, 39]. PrP^CWD^ in renal tissues has, however, been demonstrated by sPMCA [40] and it has been shown that CWD-infected cervids can shed infectious prions in urine [41–43], although the proximal source of PrP^CWD^ in urine is not known [40]. In the present study, PrP^CWD^ positive immunolabeling of kidney samples was detected along the wall of the renal arteries, especially in the main renal artery and the wall of arcuate arteries (Fig. 6a, b). In scrapie-affected sheep, prion deposition has been found in renal papillae and renal corpuscles [33, 44]. Periarterial and periarteriolar immunolabeling could be due to spread of prions through peripheral nerves [44] since the wall of these renal arteries is strongly innervated and sympathetic nerve fibers from the renal plexus enter the kidney accompanying the branches of the main renal artery. To our knowledge, this is the first description of PrP^CWD^ deposition, detected by conventional techniques, in renal arteries of CWD-infected deer.

D1 also presented intense PrP^CWD^ immunolabeling in the renal cortex associated with accumulations of inflammatory cells (Fig. 7a). Inflammatory processes affect prion pathogenesis and peripheral accumulation [45, 46], and chronic nephritis triggers prionuria in prion-infected mice [47]. In addition, PrP^CWD^ shedding has been reported in CWD infected deer presenting with inflammatory kidney disease [41]. We cannot predict the effect of prion accumulation in the arterial walls on prionuria, however, we have observed that inflammatory kidney conditions greatly increase PrP^CWD^ deposition in renal tissues from deer with CWD, which might increase shedding of PrP^CWD^ within urine. This deer also showed PrP^CWD^ accumulation in the lungs associated with inflammatory cell foci and in the bronchiolar epithelium (Fig. 7b). PrP accumulation in the lung related to inflammatory conditions and in the epithelium of the bronchioles has been previously described in scrapie-affected sheep [33, 48]. Deer in our study were housed indoors with ample access to clean food and water [18]. It is likely that free-ranging deer would be at greater risk of coincident infections and commensurate inflammation that may influence the effect of protective alleles on susceptibility to CWD, tissue colonization by CWD prions and shedding.

In organs related to excreta production, differences in PrP^CWD^ deposition were found in salivary glands between deer genotypes. PrP^d^ in salivary glands, detected by conventional techniques, has been described in scrapie-affected sheep [49] and in the serous epithelial cells of the submandibular salivary gland in experimentally infected red deer [50]. Likewise, considerable PrP^CWD^ amplifying activity, similar to that observed in brain, accumulates in salivary glands of cervids with CWD [40]. Comparison of single salivary gland sections identified PrP^CWD^ immunostaining in wt/wt or S96/wt deer but not in deer expressing H95-PrP^C^ (Table 2). Intense PrP^CWD^ immunolabeling in ganglia cells immersed in the salivary gland tissue was observed in animal D4 (Fig. 6c).

Our observations suggest that deer expressing H95-PrP^C^ have reduced centrifugal trafficking via descending nerves into peripheral tissues (i.e. salivary glands, pancreas, heart and kidney). This is consistent with previous observations in different experimental prion infections [32, 33, 36, 44, 51]. However, it has been suggested that, in initial stages of CWD infection, PrP^CWD^ may be trafficked via blood [26], and infectivity has been demonstrated in blood components [52, 53]. Therefore, we cannot exclude the hematogenous route as a complementary pathway of prion dissemination.

None of the clinically affected deer presented positive immunolabeling in any of the skeletal muscle samples evaluated, including the tongue. The absence of PrP^d^ accumulation in skeletal muscles detectable by IHC techniques has been reported in both naturally and experimentally prion infected deer [54, 55]. Nevertheless, although we did not detect PrP^CWD^ deposition in skeletal muscle samples in this experimental Wisc-1 transmission to white-tailed deer, we cannot assume the complete absence of prion accumulation. Others have demonstrated the presence of PrP^CWD^ in skeletal muscles by bioassay [56], Western Blot, PMCA and tissue-blotting [34].

The presence of prion deposits in skeletal muscles has previously been reported in neuromuscular spindles, which are highly innervated structures [33, 35] and, in CWD-infected white-tailed deer, in nerve fascicles [34]. Although most of the vagus nerve samples evaluated in the present study showed positive immunolabeling, sciatic nerve and brachial plexus samples presented sparse or no PrP^CWD^ deposits (Table 2). Our results are similar to those in mule deer naturally infected with CWD [32]. We did not detect PrP^CWD^ deposition in forelimb and hindlimb skeletal muscle samples, not even in the neuromuscular spindles. Due to the fact that brachial plexus and sciatic nerve innervate, respectively, the forelimb and hindlimb muscles, and taking into account that prions can spread to these muscles via these neural pathways [32], we can suggest that the scant or absent PrP^CWD^ immunolabeling in brachial plexus and sciatic nerve samples from deer in our study may relate to the absence of deposits in these groups of muscles.

Cumulative evidence supports the limiting effect of H95 and S96 PrP^C^ polymorphisms on natural CWD infection and disease progression [16–18, 37]. These PrP^C^ allelic variants modulate CWD propagation and the efficiency of intraspecies CWD transmission [19, 57]. The passage of CWD prions in white-tailed deer expressing the H95-PrP^C^ led to the emergence of the novel CWD strain H95^+^ [19]. This strain presented distinct biochemical and transmission properties, efficiently propagating in transgenic mice expressing deer S96-PrP^C^ and in non-transgenic C57BL/6 mice [19, 20]. Wisc-1 propagation in deer expressing the H95-PrP^C^ also presented limited peripheral PrP^CWD^ accumulation.

As demonstrated in sheep with scrapie, *PRNP* genotype strongly influences the tropism and distribution of PrP deposits [23, 25, 30]. In the present study, the observed effects of the H95 allele in prion distribution resemble those described for sheep expressing the resistance-associated allele ARR at codons 136, 154 and 171 of the prion protein. ARR/VRQ sheep, despite developing disease and accumulating PrP^Sc^ in the brain, present a much more limited and infrequent PrP^Sc^ distribution in lymphoid tissues compared to those with other susceptible genotypes [30, 58, 59]. This effect is likely due to a modulation of the prion pathogenesis [25, 60] and it is not necessarily associated with the prolonged incubation period [25].

The similarities in peripheral PrP^CWD^ accumulation between H95/wt and H95/S96 deer indicate a limiting role for the H95 amino acid substitution on the production and/or accumulation of PrP^CWD^. Similar observations have been made in goats expressing methionine at codon 142, which show reduced incidence of scrapie infection and a lower tendency to accumulate PrP^Sc^ outside the brain compared to 142 isoleucine homozygotes [50, 61]. In contrast, our findings suggest that H95-PrP^C^ does not affect LRS involvement in CWD-affected deer (Table 1).

Influences of genotype on PrP^CWD^ deposition pattern have been described in experimentally infected mule deer, with F225/S225 deer presenting with milder PrP^CWD^ accumulation and limited tissue distribution compared to S225 homozygotes at identical intervals post-inoculation. This suggests the F225 amino acid variant limits prion conversion and delays PrP^CWD^ tissue accumulation [31, 57]. Similar observations have been made in white-tailed deer expressing the S96 PrP^C^, which, compared to wt/wt deer, show reduced PrP^CWD^ in brain and lymphoid tissues, consistent with slower disease progression [17, 26]. Deer of this genotype have also been reported to present lower PrP^CWD^ immunostaining scores in the obex than wt/wt deer [62, 63]. However, observations were made in naturally infected animals in different stages of the disease. Although we observed certain differences between wt/wt and S96/wt deer in particular brain regions (Fig. 2), the overall intensity and peripheral distribution of PrP^CWD^ was similar for both genotypes at the terminal stage of the disease (Tables 1 and 2). By contrast, the H95 polymorphism was a stronger driver of disease phenotype. The interference exerted by H95-PrP^C^ in the replication and tissue accumulation of Wisc-1 prions relates to the biology of cervid PrP^C^ polymorphisms and the evolution of CWD prion strains [19, 20].

Given that the diversity of CWD agents can be expanded in deer expressing PrP^C^ polymorphisms [19, 20, 64], our findings cannot be generalized to include all potentially existing CWD strains which will likely have distinct host specific interactions. Prion disease characteristics, including the lesion distribution and the IHC phenotype of PrP^d^ accumulation, are strongly influenced by the infecting prion strain [21, 65–67]. Thus, it is possible that the reduced PrP^CWD^ immunolabeling observed in areas of the H95/S96 brain (Figs. 1 and 2) could be due to intra-species transmission barrier determined by the compatibility between the invading strain and the PrP^C^ sequence of the host [68, 69]. Likewise, heterozygous deer presented lower levels of PrP^CWD^ deposits in certain rostral brain areas, as compared to wt/wt deer (Fig. 2). These observations are consistent with the reduced amounts of PrP^CWD^ as detected by Western blottimg [18]. Deer in the present study were orally inoculated with Wisc-1 prions [19], a CWD source obtained from animals homozygous for the wt-PrP^C^ [18]. Therefore, transmission of the Wisc-1 strain into H95/S96 deer involves the adaptation of the infectious agent to this new host microenvironment, which could partially explain the reduced PrP^CWD^ accumulation in particular brain regions, considering that the expression of wt-PrP^C^ favors the propagation of the Wisc-1 strain [19].

The PrP^CWD^ accumulated in the H95/S96 animal (H95-PrP^CWD^) [19] is more PK-sensitive than PrP^CWD^ from deer with at least one wt allele [18]. Differences in PrP resistance to proteolytic degradation can lead to variable results in diagnostic tests. For example, atypical/Nor98 scrapie isolates are highly sensitive to PK digestion compared with classical scrapie strains, which leads to inconsistent diagnosis by PrP^d^ IHC [70]. Therefore, this particular characteristic of H95-PrP^CWD^ may also explain the lower immunolabeling observed in the brain of H95/S96 deer. Nevertheless, prion disease neuropathological phenotypes, which include the PrP^d^ profile, may depend on complex interactions between the infecting prion strain and host factors (e.g. the *PRNP* genotype) [23]. This host-pathogen interaction might explain the differences observed between deer genotypes with respect to the PrP^CWD^ deposition in the cerebellum (Fig. 2). However, as mentioned, other CWD strains could differentially interact with PrP primary sequence and those effects should be further explored to understand how PrP^C^ polymorphisms modulate the propagation of CWD infectious agents.

## Conclusions

The present study indicates that expression of the H95 PrP^C^ polymorphism limits the intensity and distribution of PrP^CWD^ aggregates in a wide variety of tissues and supports previous findings on the role deer *PRNP* genotype plays on the modulation of CWD progression and adaptation of CWD strains. Although breeding programs selecting for less susceptible *PRNP* genotypes can be effective in reducing scrapie prevalence in flocks [71], our data regarding the impact of deer *PRNP* genotypes need to be interpreted with caution. Animals expressing H95 PrP^C^, although presenting with a more limited prion distribution and longer incubation periods, represent the adaptation of a new strain [19]. Genetic enrichment for *H95-PRNP* alleles in deer herds may enhance the selection of H95+ or of novel strains with increased ability to propagate in genotypes including this PrP^C^ polymorphism. The significantly longer incubation periods observed in deer with *H95-PRNP* alleles may not impact secretion of CWD (i.e., less CWD secreted over longer time periods). The emergence of new CWD strains could implicate a zoonotic potential [20].

## Methods

### Animals and tissue sampling

Brain and peripheral tissues were collected from orally infected white-tailed deer expressing different *PRNP* genotypes: Q95G96/Q95G96 (wt/wt; *N* = 5; D1-D5), S96/wt (*N* = 3; D6-D8), H95/wt (*N* = 1; D9) and H95/S96 (N = 1; D10) [18]. All deer developed terminal clinical prion disease, showing different survival periods depending on their PrP^C^ primary structure. Deer were euthanized when evident clinical signs were established and persisted for a week [18]. Animals were euthanized by pentobarbital overdose (120 mg/kg) following anesthesia with a cocktail of 0.2 mg/kg medetomidine, 4.0 mg/kg ketamine and 0.2 mg/kg butorphanol. Non-infected white-tailed deer of wt/wt *PRNP* genotype (*N* = 2; D11 and D12) were also collected, euthanized as described, and examined as controls. No prion aggregates were found in any of the samples obtained from these animals.

All deer used in this study were obtained as wild-abandoned fawns (no permit was required) from the CWD-free region of northern Wisconsin, and, prior to the bioassay, all animals tested negative for CWD, as determined by tonsil biopsy [18]. This study was carried out in accordance to the recommendations in the Guide for the Care and Use of Laboratory Animals of the National Institutes of Health. The research animal ethics protocol was approved by the School of Veterinary Medicine Animal Care and Use Committee at the University of Wisconsin.

### PrP^CWD^ immunohistochemical analysis

The brain and peripheral tissues were fixed in 10% formalin and embedded in paraffin. Tissues were cut into 5-μm-thick sections and mounted on glass slides for immunohistochemical analysis. PrP^CWD^ immunolabeling was performed using the monoclonal antibody 6H4 (Prionics, Switzerland) at the dilution recommended by the manufacturer, followed by incubation with a secondary anti-mouse antibody, a peroxidase-streptavidin conjugate, a substrate chromogen and hematoxylin counterstain, as previously described [18]. Immunostaining was performed at the Wisconsin Veterinary Diagnostic Laboratory using NexES automated immunostainer (Ventana Medical Systems).

Sections were then scanned using a Hamamatsu NanoZoomer 2.0RS digital scanner (Hamamatsu Photonics, Hamamatsu, Japan).

The distribution, morphology and intensity of PrP^CWD^ deposits were blindly evaluated in 15 brain areas: Obex, cerebellar molecular layer (Cml), cerebellar Purkinje cell layer (Cpl), cerebellar granular layer (Cgl), cerebellar white matter (Cwm), Pons, superior colliculus (SC), thalamus (TH), hypothalamus (HT), caudate nucleus (CA), septal nucleus (SN), hippocampus (HC), frontal cortex grey matter (FCgm), frontal cortex white matter (FCwm) and olfactory bulb (OB). The intensity of PrP^CWD^ accumulation in each brain area was semi-quantitatively scored on a scale of 0 (absence of deposits) to 4 (severe deposition) in order to obtain a PrP^CWD^ brain profile for each genotype. Data was analyzed using GraphPad Prism version 6.0 (GraphPad Software, La Jolla, CA, USA).

The presence of PrP^CWD^ deposits was also evaluated in the following tissues: lymph nodes (retropharyngeal, submandibular, axillary, prescapular, prefemoral, popliteal, inguinal, tracheobronchial, ileocecal, hepatic, pancreatic and adrenal), spleen, third eyelid, tonsil, pituitary gland, peripheral nerves (vagus, brachial plexus and sciatic), skeletal muscle, heart, intestine, liver, pancreas, kidney, adrenal glands, lung, salivary glands (parotid, submandibular and sublingual), retina and optic nerve. PrP^CWD^ immunostaining in lymphoid tissues was scored as: - (absence of immunostaining), + (< 10% of the lymphoid follicles presenting immunostaining), ++ (10–50% of the lymphoid follicles presenting immunostaining) and +++ (> 50% of the lymphoid follicles presenting immunostaining) as previously reported [43]. For the remainder of the tissues, PrP^CWD^ immunostaining was subjectively scored as: - (absence of immunostaining), + (minimal to slight immunostaining found in a small part of the tissue section), ++ (moderate immunostaining present in several areas of the evaluated tissue section and/or several tissue sections), and +++ (strong and widespread immunostaining throughout the entire section and/or several tissue sections).

## Abbreviations

CA: Caudate nucleus
Cgl: Cerebellar granular layer
Cml: Cerebellar molecular layer
Cpl: Cerebellar Purkinje cell layer
CWD: Chronic wasting disease
Cwm: Cerebellar white matter
ENS: Enteric nervous system
FCgm: Frontal cortex grey matter
FCwm: Frontal cortex white matter
HC: Hippocampus
HT: Hypothalamus
IHC: Immunohistochemistry
LRS: Lymphoreticular system
OB: Olfactory bulb
*PRNP*: Prion protein gene
PrP^C^: Cellular prion protein
PrP^CWD^: Abnormal prion protein
PrP^d^: Deposition of abnormal prion protein
SC: Superior colliculus
SN: Septal nucleus
TH: Thalamus
TSE: Transmissible spongiform encephalopathy
Wt: Wild-type
